# Intraoperative Evaluation of Whole Sentinel Lymph Nodes Using a One-Step Nucleic Acid Amplification Assay in Endometrial Cancer: A Prospective Study

**DOI:** 10.3390/medicina61071221

**Published:** 2025-07-04

**Authors:** Shinichi Togami, Nozomi Furuzono, Mika Mizuno, Hiroaki Kobayashi

**Affiliations:** Department of Obstetrics and Gynecology, Faculty of Medicine, Kagoshima University, Kagoshima 890-8520, Japan; nozomi.fur051120@gmail.com (N.F.); mizunomizuno512@gmail.com (M.M.); k8292219@kadai.jp (H.K.)

**Keywords:** endometrial cancer, micrometastasis, one-step nucleic acid amplification, sentinel lymph node

## Abstract

*Background and Objectives*: The aim of this prospective study was to evaluate the diagnostic accuracy of the one-step nucleic acid amplification (OSNA) assay for the intraoperative assessment of sentinel lymph node (SN) metastases, including micrometastases in patients with stage IA low-grade endometrial cancer. *Materials and Methods*: A prospective analysis was conducted on 204 patients with low-risk endometrial cancer who underwent hysterectomy, bilateral salpingo-oophorectomy, and sentinel node navigation surgery. SNs were analyzed intraoperatively using the OSNA assay, and positive patients underwent systematic pelvic lymphadenectomy. *Results*: Among the 204 patients included, SN metastases were identified in 12 patients (6%), including 10 patients with micrometastases and 2 patients with macrometastases. No metastases were detected in non-SNs in any of the 12 patients. Recurrence occurred in two patients (1%), involving the vaginal stump and pelvic cavity dissemination, but no lymph node recurrence was observed. The OSNA assay identified a proportion of micrometastases in low-risk endometrial cancer. While a direct comparison with conventional pathological ultra-staging was not performed in this study, the detection rate of micrometastases appears higher than that reported in historical controls. *Conclusions*: This is the first prospective study to evaluate the intraoperative use of the OSNA assay for whole SNs in endometrial cancer. The results suggest that the OSNA assay enhances the detection of micrometastases, enabling a more accurate assessment of SN metastases. In low-risk endometrial cancer, systematic pelvic lymphadenectomy may be safely omitted in patients with SN-positive micrometastases. Further prospective studies are necessary to validate these findings and support the adoption of this approach in clinical practice.

## 1. Introduction

Endometrial cancer is the sixth most commonly diagnosed cancer among women worldwide, with 417,000 new patients and 97,000 deaths reported in 2020 [1]. In Japan, approximately 17,000 women are newly diagnosed with endometrial cancer, and approximately 2600 women die from the disease annually [2]. In the management of endometrial cancer, pelvic lymphadenectomy has been a standard procedure for accurate surgical staging because lymph node metastasis (LNM) is an important prognostic factor [3,4]. However, complications, such as lower limb lymphedema and pelvic lymphocele, remain significant concerns [5]. Recently, sentinel node (SN) mapping has been introduced as a technique in endometrial cancer [5] that allows for the accurate detection of LNM while reducing the risk of lower limb lymphedema [6,7,8,9,10], and it is now recommended in clinical guidelines [11,12].

The diagnosis of SN metastasis is usually made by slicing the SN into sections that are 2 mm apart and using hematoxylin and eosin (H&E) staining and/or immunohistochemical staining. Ultra-staging is used to diagnose microscopic LNM, such as micrometastasis and isolated tumor cells (ITCs), by undertaking further thin-sectioning (200 μm) of the excised SN and applying H&E staining and immunohistochemistry. Pathologists conduct these SN diagnoses; however, a major concern is the cost and time associated with these procedures, including ultra-staging, as well as the significant burden placed on pathologists and technicians. Furthermore, ultra-staging is inherently limited by several practical and technical challenges. These include inter-observer variability in histopathological interpretation, sampling bias due to the partial sectioning of the lymph node, and the resource-intensive nature of serial slicing and immunohistochemical staining. These factors may reduce diagnostic reproducibility and increase turnaround time, especially in high-volume clinical settings. Therefore, there is a growing need for objective, rapid, and standardized techniques for the evaluation of sentinel lymph node metastasis.

Molecular biological approaches, such as reverse transcription–polymerase chain reaction (RT-PCR), have proven useful in diagnosing LNM in various types of cancer and serve as a practical alternative to conventional pathological methods [13,14]. However, RT-PCR has the drawback of being complex, time-consuming, and costly, which poses challenges for its widespread adoption in clinical practice.

In recent years, a one-step nucleic acid amplification (OSNA) assay has been developed, utilizing the reverse transcription–loop-mediated isothermal amplification (RT-LAMP) method. This assay quantifies the copy number of cytokeratin-19 (CK19) mRNA [15,16], eliminating the need for RNA extraction and enabling the fast and straightforward amplification of target genes within 30 to 40 min.

In recent years, the molecular classification of endometrial cancer has gained increasing importance in guiding risk stratification and treatment decisions. According to The Cancer Genome Atlas (TCGA) and subsequent studies, endometrial cancers can be grouped into four molecular subtypes: POLE ultramutated, microsatellite instability-high (MSI-H), copy number-low (MMR-proficient), and copy number-high (p53 abnormal) categories [17,18]. These subtypes are associated with distinct clinical behaviors and prognoses. Notably, POLE-mutated tumors tend to have excellent outcomes and a low risk of lymph node metastasis, while p53-abnormal tumors are more aggressive and frequently exhibit nodal involvement. MSI-H- and MMR-deficient tumors may also show an intermediate risk of lymphatic spread. Although our present study did not assess molecular markers, future studies should explore their potential correlation with sentinel lymph node metastasis to refine surgical strategies [18].

The one-step nucleic acid amplification (OSNA) assay is a rapid, automated molecular diagnostic technique developed to detect sentinel lymph node (SN) metastases by quantifying cytokeratin 19 (CK19) mRNA expression. Unlike conventional histopathological analysis, OSNA analyzes the whole lymph node, thereby reducing sampling errors and improving diagnostic sensitivity. The utility of the OSNA assay has been reported across various types of cancer, and in recent years, there have been a few reports of its application in endometrial cancer [19,20,21,22]. In our multicenter prospective study evaluating the diagnostic performance of the OSNA assay for LNM in endometrial cancer, the concordance rate between the OSNA assay and conventional pathological diagnosis was 0.979, with a sensitivity and specificity of 0.918 and 0.989, respectively [23]. However, to date, no studies have reported using the OSNA assay to evaluate whole SNs intraoperatively for LNM in endometrial cancer, followed by sentinel node navigation surgery (SNNS). Additionally, the potential for non-sentinel lymph node (non-SN) metastases when LNM is diagnosed as microinvasive using the OSNA assay remains unknown. Therefore, this study aimed to assess the reliability of diagnosing whole SNs using the OSNA assay in a real-world clinical setting and to investigate the likelihood of non-SN metastases in patients diagnosed as microinvasive using the OSNA assay during SNNS.

## 2. Materials and Methods

### 2.1. Study Design and Patients

**Figure 1:**
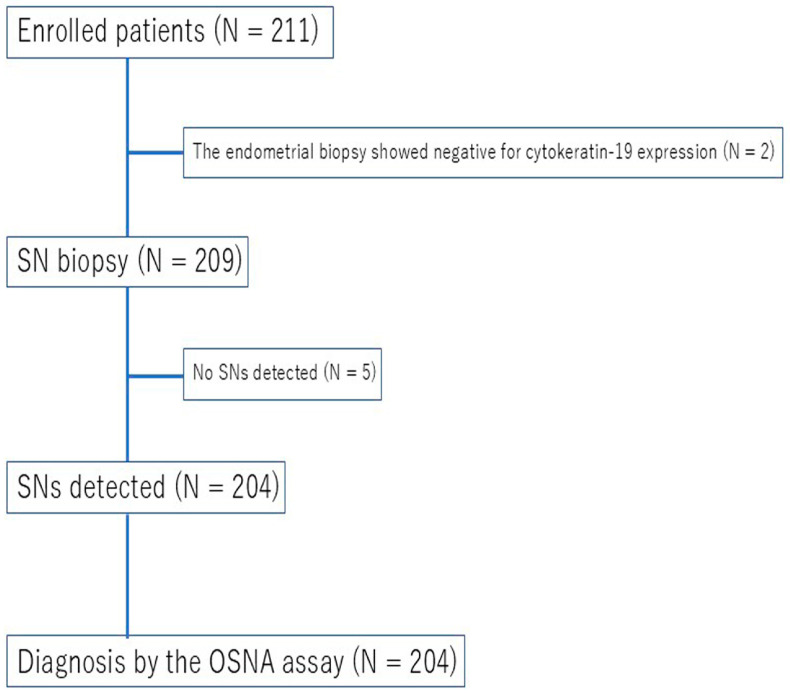
Flowchart of this prospective study.

At the Kagoshima University Hospital, patients diagnosed with stage IA endometrioid carcinoma grade 1–2 who underwent hysterectomy, bilateral salpingo-oophorectomy, and SNNS between February 2021 and September 2024 were prospectively enrolled in the clinical trial before surgery. The flowchart of this prospective study is represented in Figure 1. This prospective study was approved by the institutional review board of Kagoshima University Hospital (2020-0123), and written informed consent was obtained from all patients. Although international guidelines such as NCCN and ESGO-ESTRO-ESP do not recommend systematic lymphadenectomy for low-risk endometrial cancer, in Japan, SLN mapping is not yet covered by national health insurance. Consequently, patients with positive SNs are often managed with additional sys lymphadenectomy. In this study, systematic lymphadenectomy was performed in SN-positive cases as part of the approved institutional protocol, with a particular focus on assessing the necessity of systematic lymphadenectomy in patients with micrometastases. Patients with a low CK19 protein expression in the primary tumor tissue, those with hypersensitivity to radioactive isotopes (RI) or indocyanine green (ICG), those who received neoadjuvant chemotherapy, and patients with psychiatric disorders that could impede their participation in the trial were excluded. Patients were preoperatively diagnosed with clinical stage IA endometrioid endometrial cancer of grade 1 or 2 based on a histological examination of endometrial biopsy specimens. Radiologic staging was performed using magnetic resonance imaging (MRI) and computed tomography (CT). All imaging studies were interpreted by board-certified radiologists, and the clinical stage was assigned based on the absence of deep myometrial invasion or extrauterine disease on imaging.

SLN mapping was performed using a hybrid technique that combined the RI and ICG methods. For the RI method, a total dose of 148 MBq of technetium-99 m phytate was injected into the cervix at the 3 and 9 o’clock positions on the day prior to surgery. Planar lymphoscintigraphy and single-photon emission computed tomography/computed tomography (SPECT/CT) were then performed to anatomically localize potential SNs.

Immediately before surgery, 1.0 mL of ICG (diluted 10-fold to 0.25 mg/mL) was injected into the cervix at the same positions. SNs were visualized intraoperatively using a near-infrared fluorescence imaging system.

In most cases, both tracers identified concordant SNs. However, if a lymph node was identified by either RI or ICG alone, it was still considered an SN and resected accordingly. Therefore, modality-specific detection rates were not analyzed separately in this study.

Intraoperatively, the removed SNs were bisected along the longitudinal axis, and stamped cytology was performed on the cut surface before the whole SN tissue was assessed for metastasis using the OSNA assay. In all patients with positive SN metastasis, pelvic lymphadenectomy (PLA) was performed, and all non-SNs resected during PLA were analyzed for LNM using the OSNA assay. Additionally, a detailed analysis was conducted for each patient with positive SN metastasis, including their clinicopathological background and OSNA assay results.

Postoperative adjuvant chemotherapy (paclitaxel plus carboplatin) was administered in patients who met any of the following criteria based on the final pathological evaluation: non-endometrioid histology, a tumor grade other than G1 or G2, myometrial invasion ≥ 50%, LVSI positivity, or SN metastasis, as detected by the OSNA assay.

### 2.2. One-Step Nucleic Acid Amplification Assay

The OSNA assay was performed intraoperatively by trained laboratory technicians in a dedicated gynecologic research laboratory. While the OSNA system can analyze up to 14 lymph nodes simultaneously, in this study, 1–4 sentinel lymph nodes were typically assessed per patient. Based on routine clinical workflow, the total time required for homogenization, the amplification of CK19 mRNA, and its interpretation was approximately 30–40 min per case, as confirmed by both laboratory staff and the device manufacturer. However, individual assay durations were not formally recorded.

Diagnosis using the OSNA assay was performed following the same method as in our previous study [22,23]. To prevent RNA degradation, SNs were processed as quickly as possible after excision. The SN slices were solubilized in 4 mL of glycine buffer (glycine, 200 mmol/L; dimethyl sulfoxide, 20%; detergent, 5%; pH, 3.5) on ice using a homogenizer, followed by centrifugation at 10,000× *g* for 60 s at room temperature (1–30 °C). The solubilized lymph nodes were promptly collected from the intermediate layer. Using this “LN solubilization solution,” CK19 mRNA was amplified using the RT-LAMP method with six primers, using LS93R and a dedicated device (RD-200). Additionally, to assess the RNA quality, the OSNA system was used to evaluate the expression levels of β-actin mRNA, the results of which were reported within approximately 30–40 min. Previous studies [24] have shown that sentinel nodes are considered positive for metastasis if the CK19 mRNA copy numbers are ≥250 cCP/μL and negative if they are <250 cCP/μL. Furthermore, in line with previous research [25], positive results were categorized as micrometastasis (+) if the values were >250 and <5000 cCP/μL and as macrometastasis (++) if they were >5000 cCP/μL. No cutoff value was established for ITCs.

### 2.3. Quality Assurance and Training

All surgical procedures were performed by four gynecologic oncologists certified by the Japan Society of Obstetrics and Gynecology. Among them, two surgeons were also certified as endoscopic surgery specialists by the Japan Society of Gynecologic and Obstetric Endoscopy and Minimally Invasive Therapy. These qualifications ensured the standardization and reproducibility of the surgical and sentinel lymph node mapping techniques across all cases.

### 2.4. Postoperative Follow-Up

Postoperative surveillance was conducted according to a standardized protocol. During the first year after surgery, patients underwent follow-up examinations every 1 to 3 months, which included pelvic examinations, vaginal cuff cytology, transvaginal ultrasonography, serum tumor marker assessments, and CT scans every 6 months to detect recurrence. After the first year, follow-up intervals were extended to every 4 to 6 months, depending on individual clinical conditions, and continued for at least five years.

## 3. Results

This prospective study enrolled 211 patients, excluding two patients with negative CK19 immunohistochemistry results on endometrial biopsy and five patients where the SNs could not be identified intraoperatively; therefore, 204 patients were included in the final analysis (Figure 1). The median follow-up period was 16 months (range: 1–40 months), during which recurrence events were monitored and recorded. Table 1 summarizes the clinicopathological factors of the 204 analyzed patients. The median age and body mass index (BMI) were 57 years (range: 28–91) and 26.9 kg/m^2^ (range: 16.7–50.9), respectively. Open surgery, laparoscopy, and robotic surgery were performed on 13 patients (6%), 39 patients (19%), and 152 patients (75%), respectively. The final pathological subtypes included endometrioid carcinoma in 190 patients (93%), serous carcinoma in six patients (3%), clear cell carcinoma in five patients (3%), and other subtypes in three patients (1%). The FIGO (International Federation of Gynecology and Obstetrics) 2009 staging distribution was as follows: IA in 165 patients (81%), IB in 21 patients (10%), II in 2 patients (1%), IIIA in 3 patients (1%), IIIB in 1 patient (1%), and IIIC1 in 12 patients (6%). Lymph-vascular space involvement (LVSI) was observed in 27 patients (13%), and 30 patients (15%) exhibited myometrial invasion exceeding one-half of the myometrial thickness.

Table 2 summarizes the outcomes related to SN factors. The median number of SNs resected per patient was two (range: 1–6). Metastasis in SNs was detected in 12 patients (6%) using the OSNA method, with two patients classified as “++” and ten patients classified as “+.” Recurrence was observed in two patients (1%), with the recurrence sites being the vaginal stump in one patient and pelvic cavity dissemination in the other. No lymph node recurrences were reported.

Table 3 summarizes the clinicopathological factors and OSNA assay-related outcomes of the 12 patients with SN metastasis. The final pathological subtype was endometrioid carcinoma in 11 patients and clear cell carcinoma in 1 patient. Myometrial invasion exceeding one-half of the thickness was observed in five patients, and LVSI was positive in eight patients. Stamped cytology of the largest cross-section of the SN was positive in five patients, including both patients classified as “++” using the OSNA assay (copy numbers: 170,000 copies/μL and 49,000 copies/μL). All 12 patients underwent pelvic lymph node dissection, and no metastases were detected in non-SNs. Additionally, none of these 12 patients experienced recurrence during the follow-up period.

## 4. Discussion

The advantages of the OSNA assay include reducing the workload of pathologists and technicians, automating the diagnostic process, and enabling the accurate molecular-based detection of metastasis in whole LNs. This study is the first to report the intraoperative use of the OSNA assay to diagnose metastasis in whole SNs and perform SNNS for endometrial cancer in a clinical setting. Among the 204 patients analyzed, 12 patients (6%) were identified as SN metastasis-positive (2 patients with ++ and 10 with +). No LN recurrence was observed, demonstrating that the OSNA assay can be effectively applied in clinical practice while leveraging its inherent advantages.

The utility of the OSNA assay was first reported by Tsujimoto et al. for diagnosing metastatic LNs in breast cancer [16]. In this report, the OSNA assay was concluded to have excellent diagnostic performance for LNMs across various cancer types. While conventional histopathological ultra-staging is considered a reliable method for SN evaluation, it is labor-intensive, subject to inter-observer variability, and may miss micrometastases due to sectioning limitations. In contrast, the OSNA assay allows the standardized molecular-based evaluation of the entire LN tissue, potentially increasing detection accuracy and consistency. Furthermore, the diagnostic performance of the OSNA assay has been previously validated in a multicenter setting. In our multicenter study comparing the diagnostic performance of the OSNA assay and the conventional pathological diagnosis for LNM in endometrial and cervical cancers, we reported a concordance rate of 0.979 (95% confidence interval [CI]: 0.961–0.991) between the histopathological diagnosis and the OSNA assay. The sensitivity and specificity of the OSNA assay were 0.918 (95% CI: 0.819–0.973) and 0.989 (95% CI: 0.973–0.997), respectively. Additionally, other studies comparing the OSNA assay and conventional pathological diagnosis for endometrial cancer have reported concordance rates, sensitivities, and specificities of 83–99%, 50–100%, and 82–100%, respectively [19,21,26,27]. These findings align with our previous study, suggesting that the OSNA assay demonstrates excellent diagnostic performance for detecting LNM.

We performed SNNS for patients preoperatively diagnosed as having FIGO stage IA endometrioid adenocarcinoma, grade 1 or 2. In this study, 81% of patients were confirmed to have postoperative FIGO stage IA (2009 classification). These patients typically belong to a low-risk group for SN metastasis; however, intraoperative analysis using the OSNA assay identified SN metastasis in 12 patients (6%). In our previous multicenter study, the rate of LNM detected using the OSNA assay in endometrial cancer was 11.6% [23]. The Spanish multicenter study [19], ENDO-OSNA, reported an SN metastasis rate of 19.7% using the OSNA assay. Similarly, Kosťun et al. [24] reported an SN metastasis rate of 20.7% using the OSNA assay. The higher rates of SN metastasis observed in these studies may be attributed to the inclusion of patients at the low-risk FIGO stage IA and those with disease stages II and III. These broader inclusion criteria likely contributed to the increased SN metastasis detection rates. The variation in reported sentinel lymph node (SN) metastasis rates across different studies may be attributed to several factors. These include differences in patient selection criteria, such as the inclusion of high-risk histologic subtypes, tumor grade, and the depth of myometrial invasion. Additionally, variations in surgical techniques, SN mapping methods (e.g., the use of indocyanine green vs. other tracers), and pathological evaluation protocols (including the number of sectioned slices and the use of immunohistochemistry) may also contribute to this discrepancy. Furthermore, studies utilizing the OSNA assay may report higher detection rates of micrometastases due to its high sensitivity and whole-node analysis compared to conventional histopathological methods, which rely on partial tissue sampling.

In the current study, SN metastasis detected using the OSNA assay consisted of macrometastases in two patients (1%) and micrometastases in ten patients (4.9%). In the ENDO-OSNA trial [19], the rate of SN metastases detected using the OSNA assay was 4.8% for macrometastases and 14.9% for micrometastases, whereas histopathological analysis identified 5.4% macrometastases and 3.4% micrometastases, demonstrating a higher detection rate of micrometastases using the OSNA assay. Similarly, Fera et al. [21] reported that in endometrial cancer, the OSNA assay identified SN metastases as macrometastases in 0.7% of cases and micrometastases in 2.5% of cases, showing a comparable trend between higher micrometastasis detection rates and the OSNA assay. Fanfani et al. [28] reported that the detection rate of micrometastases using the OSNA assay was higher than that observed with pathological ultra-staging in their cohort. However, the authors also noted that this difference may reflect a relatively low number of node-positive cases and the OSNA cut-off value, recommending further validation in prospective studies. These findings, consistent with our study, suggest that the OSNA assay is particularly effective in detecting micrometastases in the SNs of endometrial cancer, highlighting its utility for accurate LNM evaluation.

In this study, recurrence was observed in two patients (1%), with recurrence sites identified as the vaginal stump and pelvic cavity dissemination. No LN recurrence was detected. To the best of our knowledge, no previous studies on using the OSNA assay in endometrial cancer have reported the recurrence outcomes of the intraoperative diagnosis of whole SN using the OSNA assay. However, given that most SN metastases in low-risk endometrial cancer, as examined in this study, were micrometastases, we believe that the risk of LN recurrence associated with the intraoperative diagnosis of whole SN using the OSNA assay is considerably low.

Additionally, comparing the OSNA assay with conventional radioisotope (RI)-guided SLN biopsy methods may help clarify its relative diagnostic performance and clinical applicability. Prospective comparative studies evaluating sensitivity, specificity, and logistical feasibility between these modalities are warranted. The utility of the intraoperative OSNA assay for the diagnosis of whole SNs has been extensively reported in breast cancer [29,30]; however, no such reports exist for endometrial cancer. In breast cancer studies, the diagnoses of micrometastases using the OSNA assay have demonstrated the absence of metastases in non-SNs retrieved during axillary LN dissection, suggesting the potential to omit extensive lymphadenectomy. In our study, among the 12 patients with SN metastases, micrometastases were observed in 10 patients. None of these patients exhibited metastases in non-SNs. These findings indicate that, in low-risk endometrial cancer, where the overall frequency of LNM is low, the intraoperative OSNA assay of whole SNs could potentially allow for the omission of systematic PLA even in patients that are SN-positive, provided that the metastases are classified as micrometastases. These findings may also have implications for surgical decision-making. In patients preoperatively classified as having a low-risk, intraoperative OSNA diagnosis of SNs, this may enable the omission of full pelvic lymphadenectomy when micrometastases alone are detected, thereby reducing surgical morbidity. Moreover, sentinel lymph node (SLN) mapping has been associated with reduced surgical morbidity and better quality of life (QoL) compared to systematic lymphadenectomy. Recent studies have reported significantly lower rates of lymphedema, shorter operative times, and fewer postoperative complications in patients undergoing SLN biopsy alone without compromising on oncological safety [31,32,33]. While our study did not evaluate QoL metrics using standardized questionnaires, the adoption of SLN mapping in endometrial cancer may offer not only clinical but also patient-reported benefits in future implementation. From a practical standpoint, the OSNA assay was performed intraoperatively by trained laboratory technicians within our gynecologic research unit, independent of the pathology department. The system can process up to 14 nodes simultaneously; however, since most endometrial cancer patients present with 1–4 SNs, the time burden is minimal. Although the precise assay time was not recorded for each patient, based on institutional experience and manufacturer guidance, the total processing time—including tissue homogenization, CK19 mRNA amplification, and the interpretation of the results—was typically within 30–40 min. This operational efficiency did not interfere with surgical workflow and supports the feasibility of OSNA implementation even in moderately high-volume centers.

Given the growing emphasis on molecular classification in endometrial cancer, markers such as POLE mutations, p53 abnormality, and mismatch repair (MMR) status are increasingly recognized as predictive of recurrence and lymphatic spread. Although our study did not incorporate molecular profiling, future protocols should integrate these biomarkers to explore associations between molecular subtypes and SN metastasis.

This study has several limitations. First, the number of patients with sentinel lymph node (SN) metastases was relatively small (*n* = 12), limiting the statistical power for sub-group analyses. Second, molecular classification (e.g., POLE mutations, p53 abnormalities, and mismatch repair status) was not assessed, as it was not part of the original study protocol. Given the increasing relevance of molecular profiling in endometrial cancer, future studies should incorporate these biomarkers to enhance risk stratification and inform treatment decisions. Third, although all patients were preoperatively diagnosed with endometrioid carcinoma based on endometrial biopsy, some were found to have either serous or clear cell carcinoma on the final pathology. This reflects a limitation of biopsy-based histologic assessments, which may fail to capture the full tumor heterogeneity. Fourth, while the intraoperative OSNA assay was completed in approximately 30–40 min per case, we did not record the actual time required in each procedure. Lastly, this study did not assess cost-effectiveness. Since SLN mapping and OSNA are not yet reimbursed under Japan’s national health insurance system, costs were not billed to patients, and a formal economic evaluation was not feasible. Furthermore, although recent studies have demonstrated that the OSNA assay is more cost-effective than ultra-staging due to a reduced processing time and comparable diagnostic performance [34], such analyses could not be conducted in our study due to the current lack of reimbursement infrastructure in Japan. Nevertheless, the potential for reduced pathologist labor and omitted lymphadenectomy may offer long-term cost benefits, which should be formally assessed in future cost-effectiveness analyses. Additionally, selection bias cannot be excluded, as the study population consisted of patients treated at a limited number of institutions under specific eligibility criteria. Therefore, the generalizability of the results to broader populations or healthcare systems may be limited. Moreover, although patients were followed for a minimum of five years, the current analysis focuses on early outcomes, and long-term data are still being accumulated. These limitations should be addressed in future prospective multicenter studies.

## 5. Conclusions

In conclusion, this study is the first to evaluate prospectively the utility of the intraoperative OSNA assay for diagnosing whole SNs in endometrial cancer. Our findings suggest that the OSNA assay enables the sensitive intraoperative detection of micrometastases and facilitates accurate sentinel lymph node staging in endometrial cancer. Furthermore, in low-risk endometrial cancer, our findings suggest that the intraoperative identification of micrometastases by the OSNA assay may potentially inform decisions regarding the omission of systematic PLA. However, given current clinical guidelines and the limited number of SN-positive cases included in this study, this hypothesis should be interpreted with caution and warrants validation in future prospective multicenter studies.

## Figures and Tables

**Table 1:** Clinicopathological characteristics.

	*n* = 204
Median age (years)	57 (28–91)
Median BMI ^a^ (kg/m^2^)	26.9 (16.7–50.9)
Surgical procedure	
Open	13 (6%)
Laparoscopy	39 (19%)
Robot	152 (75%)
Final pathology	
Endometrioid	190 (93%)
Grade 1	163
Grade 2	23
Grade 3	4
Serous	6 (3%)
Clear cell	5 (3%)
Other	3 (1%)
FIGO ^b^ stage (2009)	
IA	165 (81%)
IB	21 (10%)
II	2 (1%)
IIIA	3 (1%)
IIIB	1 (1%)
IIIC1	12 (6%)
LVSI ^c^	
No	177 (87%)
Yes	27 (13%)
Myometrial invasion	
None	3 (1%)
<1/2	171 (84%)
1/2≤	30 (15%)

^a^ BMI: body mass index, ^b^ FIGO: International Federation of Gynecology and Obstetrics, ^c^ LVSI: Lymph-vascular space involvement.

**Table 2:** Sentinel lymph node-related outcomes.

	Patients (*n* = 204)
Median number of SNs removed (range)	2 (1–6)
SN metastasis	
Negative	192 (94%)
Positive	12 (6%)
++	2
+	10
Recurrence	
No	202 (99%)
Yes	2 (1%)
Vaginal stump	1
Pelvic cavity dissemination	1

SN: sentinel lymph node.

**Table 3:** Clinicopathological analysis of patients with sentinel lymph node (SN) metastasis.

	**Patient 1**	**Patient 2**	**Patient 3**	**Patient 4**	**Patient 5**	**Patient 6**	**Patient 7**	**Patient 8**	**Patient 9**	**Patient 10**	**Patient 11**	**Patient 12**
Final pathology	Endometrioid	Endometrioid	Endometrioid	Endometrioid	Endometrioid	Endometrioid	Endometrioid	Endometrioid	Endometrioid	Clear cell	Endometrioid	Endometrioid
Grade	2	1	1	1	1	2	1	2	1	-	1	1
Myometrial invasion	<1/2	1/2≤	1/2≤	1/2≤	<1/2	<1/2	1/2≤	<1/2	<1/2	1/2≤	<1/2	<1/2
LVSI	No	Yes	Yes	Yes	Yes	Yes	No	Yes	No	Yes	Yes	No
SN location	External iliac	Obturator	Obturator	External iliac	External iliac	External iliac	Obturator	External iliac	External iliac	Obturator	Obturator	Obturator
Copy number [copies/μL]	1400	980	5100	170,000	920	1100	8400	1100	1200	49,000	7400	930
Stamped cytology	Negative	Positive	Positive	Positive	Negative	Negative	Positive	Negative	Negative	Positive	Negative	Negative
Non-SN metastasis	No	No	No	No	No	No	No	No	No	No	No	No
Recurrence	No	No	No	No	No	No	No	No	No	No	No	No

## Data Availability

The original contributions presented in this study are included in the article. Further inquiries can be directed to the corresponding author(s).

## Abbreviations

The following abbreviations are used in this manuscript: body mass index (BMI), cytokeratin-19 (CK19), hematoxylin and eosin (H&E), indocyanine green (ICG), isolated tumor cells (ITCs), lymph node (LN), lymph node metastasis (LNM), lymph-vascular space involvement (LVSI), one-step nucleic acid amplification (OSNA) assay, pelvic lymphadenectomy (PLA), radioactive isotopes (RIs), reverse transcription–polymerase chain reaction (RT-PCR), reverse transcription–loop-mediated isothermal amplification (RT-LAMP), sentinel lymph node (SN), sentinel node navigation surgery (SNNS).
